# Image-Quality Assessment of Polyenergetic and Virtual Monoenergetic Reconstructions of Unenhanced CT Scans of the Head: Initial Experiences with the First Photon-Counting CT Approved for Clinical Use

**DOI:** 10.3390/diagnostics12020265

**Published:** 2022-01-21

**Authors:** Arwed Elias Michael, Jan Boriesosdick, Denise Schoenbeck, Matthias Michael Woeltjen, Saher Saeed, Jan Robert Kroeger, Sebastian Horstmeier, Simon Lennartz, Jan Borggrefe, Julius Henning Niehoff

**Affiliations:** 1 Department of Radiology, Neuroradiology and Nuclear Medicine, Johannes Wesling University Hospital, Ruhr University Bochum, 44801 Bochum, Germany; Jan.Boriesosdick@muehlenkreiskliniken.de (J.B.); Denise.Schoenbeck@muehlenkreiskliniken.de (D.S.); MatthiasMichael.Woeltjen@muehlenkreiskliniken.de (M.M.W.); Saher.Saeed@muehlenkreiskliniken.de (S.S.); JanRobert.Kroeger@muehlenkreiskliniken.de (J.R.K.); Sebastian.Horstmeier@muehlenkreiskliniken.de (S.H.); Jan.Borggrefe@muehlenkreiskliniken.de (J.B.); Julius.Niehoff@muehlenkreiskliniken.de (J.H.N.); 2 Department of Diagnostic and Interventional Radiology, Faculty of Medicine, University Hospital Cologne, University of Cologne, 50923 Cologne, Germany; simon.lennartz@uk-koeln.de

**Keywords:** photon-counting computed tomography, photon-counting detector, unenhanced CT of the head, image quality, neuroradiology, virtual monoenergetic imaging

## Abstract

In 2021, the first clinical photon-counting CT (PCCT) was introduced. The purpose of this study is to evaluate the image quality of polyenergetic and virtual monoenergetic reconstructions in unenhanced PCCTs of the head. A total of 49 consecutive patients with unenhanced PCCTs of the head were retrospectively included. The signals ± standard deviations of the gray and white matter were measured at three different locations in axial slices, and a measure of the artifacts below the cranial calvaria and in the posterior fossa between the petrous bones was also obtained. The signal-to-noise ratios (SNRs) and contrast-to-noise ratios (CNRs) were calculated for all reconstructions. In terms of the SNRs and CNRs, the polyenergetic reconstruction is superior to all virtual monoenergetic reconstructions (*p* < 0.001). In the MERs, the highest SNR is found in the 70 keV MER, and the highest CNR is in the 65 keV MER. In terms of artifacts below the cranial calvaria and in the posterior fossa, certain MERs are superior to polyenergetic reconstruction (*p* < 0.001). The PCCT provided excellent image contrast and low-noise profiles for the differentiation of the grey and white matter. Only the artifacts below the calvarium and in the posterior fossa still underperform, which is attributable to the lack of an artifact reduction algorithm in image postprocessing. It is conceivable that the usual improvements in image postprocessing, especially with regard to glaring artifacts, will lead to further improvements in image quality.

## 1. Introduction

Cranial-computed tomography (CCT) is one of the outstanding developments in medicine, which has revolutionized diagnostics and thus clinical decision making [1]. It is the most important method for the rapid examination of patients with craniocerebral trauma in cerebrovascular events, e.g., cerebral infarction or cerebral hemorrhage, and it also plays an important role in the visualization of structural changes, e.g., space-occupying lesions and degenerative brain diseases [2]. In emergency medicine, imaging is indispensable after brain trauma [3]. Regarding acute cerebrovascular events, CCT is also considered a fast and very reliable examination method, which allows an initial assessment and especially, the differentiation between ischemia and hemorrhage [4]. However, in comparison to MRI, the low contrast between gray and white matter of brain tissue remains a challenge in CCT imaging. However, the differentiation of gray and white matter is fundamental for brain diagnostics.

In 2021, the first photon-counting CT approved for clinical use (Naeotom Alpha, Siemens Healthineers, Erlangen, Germany) was introduced to the market [5]. The introduction of photon-counting detectors (PCDs) is the latest revolutionary development in clinical-computed tomography [6]. In conventional CT detectors—also called energy-integrating detectors (EIDs)—the X-ray radiation reaching the detector is first converted into visible light in the top layer of the detector, the scintillator. In the second step, this light is absorbed by a photodiode and thus, converted into an electrical signal that is proportional to the total energy that reaches the detector in a measurement interval [7]. In the photon-counting detector, however, the x-ray radiation is absorbed not by a scintillator, but directly by a semiconductor diode to which a strong voltage is applied. Each absorbed x-ray photon provides an electrical signal whose magnitude is proportional to the energy of the absorbed photon; the number of electrical signals, i.e., the number of absorbed photons, and their energy are registered in the detector [7]. Based on these technological advances, it is conceivable that PCDs provide data with higher spatial resolution and lower noise than EIDs, further providing inherent spectral information [8].

Since the introduction of the dual-energy CT (DECT) [9] into clinical diagnostics, it is possible to generate virtual monoenergetic reconstructions (MERs) using special postprocessing algorithms in addition to the conventional polyenergetic reconstruction (PER), which integrates all spectra of the X-ray beam to create an image [10]. These MERs are calculated to correspond to an image that would be created using a monoenergetic X-ray beam [11]. The construction of MERs is possible over a wide range of energies; these are expressed in kiloelectron volts (keV) [12]. Different MERs can be used to exploit the strengths of different energy levels for imaging; low keV levels provide higher soft-tissue contrast and improved imaging of the iodine-containing contrast agent [10], and higher keV levels can be used to reduce beam-hardening artifacts in particular [13]. With regard to unenhanced computed tomography of the head, the image quality of PERs and MERs has been investigated in DECTs [14].

From studies with a preclinical prototype PCCT, it is known that the quality of images from a PCD is higher than with a conventional EID [15]. However, it is unclear, if and to what extent, manufacturer claims translate into clinical routines, especially in view of the latest developments in dual-energy computed tomography. Furthermore, it is necessary to evaluate based on objective image-quality tests of which image reconstructions should be preferably used in clinical routines. Therefore, we evaluate the image quality of unenhanced CT examinations of the head performed with the first clinically approved PCCT by comparing objective image properties of polyenergetic and virtual monoenergetic reconstructions. In reference to earlier studies, the focus is set on the signal-to-noise ratio of gray and white matter, the contrast-to-noise ratio, and on indices for the extent of beam-hardening artifacts under the cranial calvaria and in the posterior fossa. Finally, these parameters will be discussed and compared with earlier results from a study of image quality in dual-layer detector CTs [14].

## 2. Materials and Methods

### 2.1. Patient Population

Institutional review board approval was obtained. Informed consent was waived due to the retrospective study design. We retrospectively identified all consecutive patients who had received an unenhanced PCCT of the head in our institute in September and October 2021. The scans were performed for diagnostic use with clinical standard protocols. To avoid additional confounders, all patients with an acute intracranial pathology diagnosed in the CT examination as well as chronic pathologies (e. g. infarcts, intracranial hemorrhages, intracranial masses, any form of edema, profound leukoencephalopathy and implants) were excluded. Patient-specific data were anonymized.

### 2.2. CT Protocols and Image Acquisition

**Figure 1:**
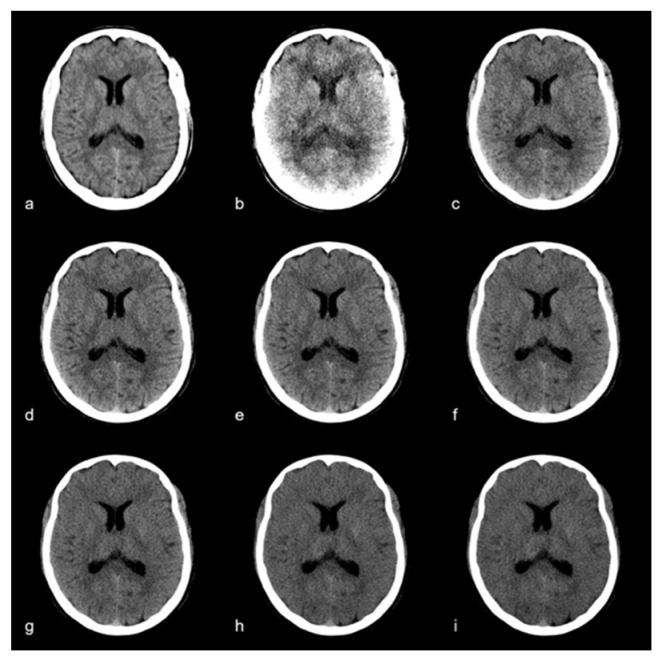
Polyenergetic reconstruction (**a**) and monoenergetic images at (**b**) 40 keV, (**c**) 60 keV, (**d**) 65 keV, (**e**) 70 keV, (**f**) 75 keV, (**g**) 80 keV, (**h**) 100 keV, (**i**) 120 keV. Window settings were kept equal for comparability.

All CT scans were performed using the clinical-approved photon-counting CT (Naeotom Alpha, software version Syngo CT VA40, Siemens Healthineers, Erlangen, Germany) with a predefined spiral CT protocol. All patients were examined in the supine position with moderate flexion in the cervical spine to perform axial acquisition in the orbitomeatal plane. Single collimation was 0.4 mm, total collimation was 57.6 mm, and pitch factor was 0.55 with a rotation time of 0.5 s. Tube voltage was 120 kVp, and tube current was modulated due to the manufacturer’s program of dose modulation. The matrix size was 512 × 512, and the field of view (FOV) was optimized for the respective head size. The reconstruction kernel HR36 was used for the PER and the kernel QR36 for the MER. The manufacturer-specific spectral workstation (Syngo.Via, VB60 version, Siemens Healthineers, Erlangen, Germany) was used to analyze the datasets. The polyenergetic and monoenergetic reconstructions were calculated from the same dataset using the manufacturer’s own algorithm; slice thickness was 3 m, and the slice increment was 3 mm. A second iteration level (Q2) was used for all reconstructions, and the current clinically used beam-hardening reduction algorithm was automatically applied to the MER; this algorithm is currently not available for the PER. The virtual monoenergetic reconstructions were made from 40 keV to 120 keV in 5 keV steps (PER and exemplary MER are shown in Figure 1).

### 2.3. Quantitative Image Analysis

**Figure 2:**
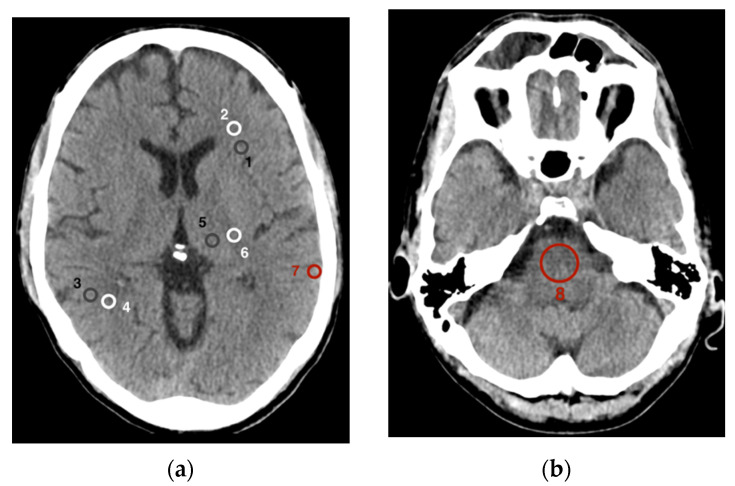
Placement of the regions of interest (**a**) at the level of the basal ganglia: frontal cortex (1) and adjacent white matter (2), parietal cortex (3) and adjacent parietal white matter (4), thalamus (5) and posterior internal capsule (6), gray matter immediately below the cranial calvaria (7); (**b**) at the level between the petrous bones, centrally in the pons (8).

Eight different regions of interest (ROIs) were set as the basis for data collection to assess image quality (Figure 2). Six of these ROIs addressed the gray and white matter and were analogous to the methods described by Neuhaus et al. [14]. For this purpose, ROIs were placed in an axial plane at the level of the basal ganglia in the frontal cortex (1) as well as in the adjacent white matter (2), in the parietal cortex (3) as well as in the adjacent parietal white matter (4), and finally in the gray matter of the thalamus (5) as well as in the white matter of the posterior internal capsule (6). A seventh ROI was placed temporoparietally in the cortical gray matter immediately below the cranial calvaria (7). At last, the eighth ROI was placed centrally in the pons (8) in an axial plane at the level between the petrous bones with the posterior fossa imaged.

Both the sizes and positions of the ROIs were accurately maintained within a CT scan in the polyenergetic and in all virtual monoenergetic reconstructions. The area of ROIs one to seven was set at 25 mm^2^ and was only minimally reduced in ROIs one and three in a few cases to truly capture only gray cortical matter and not adjacent white matter or cerebral liquor. The size of ROI eight in the pons was kept constant at 200 mm^2^. For the evaluation of signal and noise in the gray matter, the ROIs in the frontal, parietal, and thalamic gray matter were averaged together; similarly, for the white matter examination, the ROIs in the frontal, parietal white matter and the internal capsule were pooled in the same manner.

For comparability and thus, in accordance with other studies on this topic, parameters for assessing image quality were determined as described earlier [14,16]. The signal of an ROI was defined as the mean density/attenuation in Hounsfield units (HU). Noise was defined as the standard deviation (SD) of a ROI in HU. The SD of the ROI in the pons between the petrous bones is also referred to as the posterior fossa artifact index (PFAI) [17]. To also represent a measure of the artifacts and the resulting noise below the cranial calvaria, the SD of the ROI in the gray matter immediately below the calvaria is understood as the subcalvarial artifact index (SAI) [14]. In addition, the difference in attenuation between the substance affected by hardening artifacts and the adjacent same substance without hardening artifacts was determined following the approach of Zhao et al. [18].The signal-to-noise ratio (SNR) was calculated by dividing the mean density of the ROI (signal) by the associated SD of the ROI (noise). The contrast-to-noise ratio was calculated as the quotient of the difference of the mean density of two adjacent ROIs with gray matter (GM) as well as white matter (WM) and the square root of the sum of the variance of both ROIs [19]:

$$\mathrm{CNR}=\frac{\left|{\;\mathrm{mean}}_{\mathrm{GM}}-{\;\mathrm{mean}}_{\mathrm{WM}}\;\right|}{\sqrt{{\mathrm{SD}}_{\mathrm{GM}}^{2}+{\;\mathrm{SD}}_{\mathrm{WM}}^{2}}}$$

### 2.4. Statistical Analysis

Data processing and statistical analyses were performed using the statistical software R and RStudio (R Core Team (2021)). R is a language and environment for statistical computing. R Foundation for Statistical Computing, Vienna, Austria. URL https://www.R-project.org/; RStudio Version 1.4.1106, (accessed on 4 November 2021). The Shapiro–Wilk test was applied to test for normal distribution. Differences of normally distributed variables were tested using the two-sided paired *t*-test. The two-sided Wilcoxon signed-rank test was applied for testing differences in not normally distributed variables. *p*-values ≤ 0.05 were considered as statistically significant. All data are presented as mean ± SD of the mean, if not stated otherwise.

## 3. Results

A total number of 318 CCTs were reviewed in this study. After exclusion of 269 studies following the above exclusion criteria, 49 CCTs could be included in the analyses. The average tube current was 296.5 mAs. The average CTDI (computed tomography dose index) was 44.07 mGy (SD = 3.23 mGy, min = 39.4 mGy, max = 52.4 mGy) with an average scan length of 16.3 cm (SD = 0.7 cm, min = 14.8 cm, max = 17.7 cm). The average DLP (dose length product) was 718 mGy·cm (SD = 72 mGy·cm, min = 605 mGy·cm, max = 880 mGy·cm).

### 3.1. Signal and Noise in Gray and White Matter

**Figure 3:**
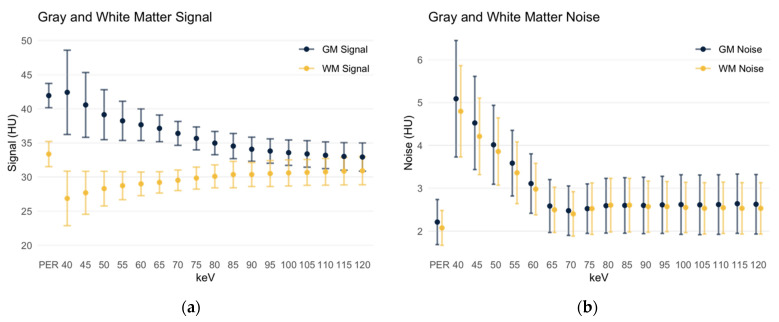
Signal and noise of gray and white matter. Data are depicted as mean ± standard deviation. GM: gray matter; WM: white matter; PER: polyenergetic reconstruction; HU: Hounsfield units. (**a**) Gray and white matter signal; (**b**) gray and white matter noise.

The gray matter signal (Figure 3a) in polyenergetic reconstructions (PERs) was 41.93 ± 1.78 HU. This signal strength was not significantly different (*p* = 0.54) from the gray matter signal in the virtual monoenergetic reconstruction (MER) at 40 keV (42.41 ± 6.17 HU). As the keV level in the monoenergetic reconstruction increases, the gray matter attenuation decreased steadily. At 45 keV grey matter attenuation was 40.56 ± 4.74 HU, and at 120 keV it is 32.93 ± 2.07 HU. Starting with the 45 keV MER, the signal in the PER was significantly higher than in the MER (*p* < 0.001).

The white matter signal (Figure 3a) in the PER was 33.37 ± 1.84 HU and significantly higher than in all monoenergetic reconstructions (*p* < 0.001). In the low keV MERs, the white matter attenuation increased steadily, resulting in 26.87 ± 3.99 HU in the 40 keV MER. With increasing keV levels, the attenuation also peaked to a maximum of 30.92 ± 2.05 HU in the 120 keV MER. Even in the 120 keV MER, the white matter signal was still significantly lower compared to the PER (*p* < 0.001).

Thus, the difference in the gray and white matter signal averages 8.56 HU in the PER and ranges from 15.54 HU in the 40 keV MER to 2.01 HU in the 120 keV MER.

The noise in the gray matter (Figure 3b) is significantly lower in the polyenergetic reconstruction (2.21 ± 0.53 HU) compared to all monoenergetic reconstructions (*p* < 0.001). In the 40 keV MER, it measures 5.09 ± 1.36 HU; the noise then decreases with increasing keV level. However, it remains 2.63 ± 0.69 HU in the 120 keV MER. Similarly, it is observed for the noise in the white matter (Figure 3b) that, again, it is significantly lower in the polyenergetic reconstruction (2.07 ± 0.41 HU) than in all monoenergetic reconstructions. In the MER levels it is higher at low keV levels (4.8 ± 1.07 HU at 40 keV), then decreases steadily to 2.53 ± 0.6 HU in the 120 keV MER.

### 3.2. Signal-to-Noise-Ratio of Gray and White Matter

**Figure 4:**
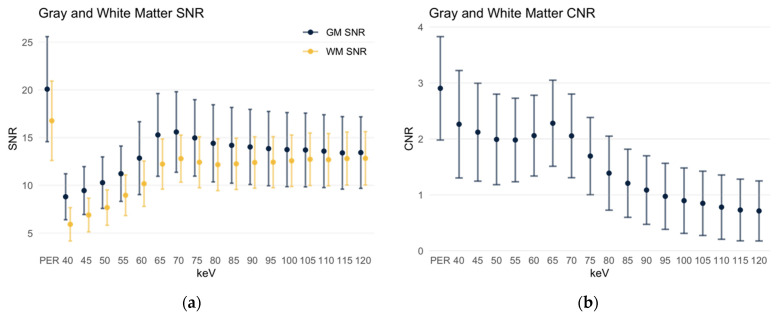
SNR and CNR of gray and white matter. Data are depicted as mean ± standard deviation. PER: polyenergetic reconstruction. (**a**) SNR of gray and white matter; (**b**) CNR of gray and white matter.

The gray matter signal-to-noise ratio (SNR, Figure 4a) in the polyenergetic reconstruction was 20.08 ± 5.50, which is significant and considerably higher (*p* < 0.001) compared to all monoenergetic reconstructions. In the MERs, SNR increased starting from 40 keV (8.81 ± 2.41) to a maximum of 15.59 ± 4.22 in the 70 keV MER. It then decreased again steadily to 13.44 ± 3.75 in the 120 keV MER.

An overall similar distribution slightly shifted towards lower values is obtained for the SNR of the white matter (Figure 4a). Again, it was significantly higher in the polyenergetic reconstruction at 16.77 ± 4.16 compared to all monoenergetic reconstructions. In the latter, it started in the 40 keV MER at 5.93 ± 4.16, then increased to a maximum in the 70 keV MER at 12.8 ± 2.47; in MERs with higher keV levels, it first dropped slightly, then increased to a second maximum in the 120 keV MER at 12.83 ± 2.8.

The contrast-to-noise ratio (CNR) of gray to white matter was also significantly higher in the polyenergetic reconstruction at 2.90 ± 0.92 (*p* < 0.001) compared to all monoenergetic reconstructions (Figure 4b). In the 40 keV MER, it was 2.26 ± 0.96, then drops briefly but reaches the maximum value of all MERs at 2.28 ± 0.77 in the 65 keV MER. With higher keV levels, the CNR then decreased steadily, in the 120 keV MER it is as low as 0.71 ± 0.54.

### 3.3. Subcalvarial and Posterior Fossa Artifact Indices

**Figure 5:**
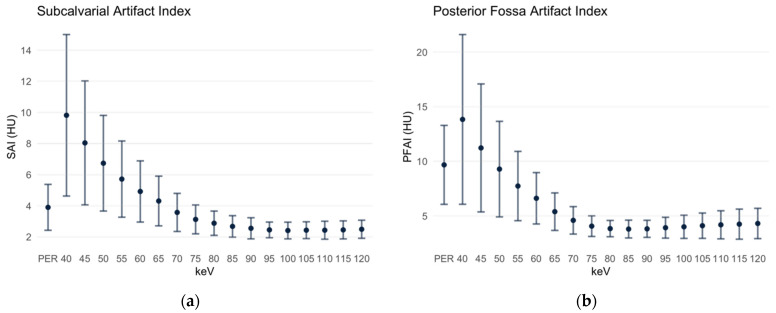
Subcalvarial and posterior fossa artifact indices. Data are depicted as mean ± standard deviation. PER: polyenergetic reconstruction. (**a**) SAI: subcalvarial artifact index; (**b**) PFAI: posterior fossa artifact index.

The SAI is defined as the standard deviation of the ROI in the gray matter immediately beneath the cranial calvaria. In the PER, it measured 3.90 ± 1.48 HU. In the MER with the low keV level, it was significantly higher at first (40 keV MER 9.82 ± 5.19 HU) but then decreased; the SAI of the 65 keV MER (4.31 ± 1.6 HU) and of the 70 keV MER (3.57 ± 1.22 HU) were not significantly different from the SAI in the PER. However, in the MER with keV levels ≥ 75 keV, the SAI is significantly lower (Figure 5a). The beam-hardening artifacts increased the density values of the tissues below the calvaria in the MER; the difference between the gray matter signal below the calvaria and the parietal gray matter was 27.04 HU (72.14 HU subcalvarial, 45.1 HU parietal, *p* < 0.001) at the maximum in the 40 keV MER, then became smaller with the increasing keV level, reaching the minimum in the 120 keV MER at 1.49 HU (34.12 HU subcalvarial, 32.63 HU parietal). In the PER, however, the gray matter signal below the calvaria was lower than the parietal, the difference being 4.08 HU (38.37 HU subcalvarial, 42.45 HU parietal, *p* < 0.001).

A similar distribution was obtained for the PFAI (Figure 5b), which was defined as the SD of ROI in the pons between the petrous bones. In the PER, the PFAI is 9.67 ± 3.61 HU. In the monoenergetic reconstructions at 40 and 45 keV, respectively, the PFAI was still significantly higher compared to the PER. No significant difference was found at 50 keV with 9.29 ± 4.37 HU (*p* = 0.186). Beginning with the 55 keV MER, the PFAI was significantly lower in all MERs with lower keV levels than in the PER; it reached its minimum in the 85 keV MER with 3.8 ± 0.82 HU. With the increasing keV level, the PFAI increases slightly; in the 120 keV MER it measures 4.31 ± 1.39 HU.

In the posterior fossa, the hardening artifacts caused a decrease in the density values in the PER (32.84 HU in the white matter of the internal capsule compared to 25.78 HU in the pons, *p* < 0.001) and in the MERs in the keV levels 40 to 60. The minimum difference was reached in the 65 keV level, where the signal in the pons was then slightly higher than in the white matter not affected by beam-hardening artifacts (28.88 HU in the white matter of the internal capsule compared to 28.98 HU in the pons, difference 0.10 HU, *p* = 0.404). In the higher keV levels this difference increased up to 1.24 HU in the 120 keV level.

The comparison of PERs and MERs is summarized in Table 1.

## 4. Discussion

This study evaluated the PER and MER image quality of unenhanced CT examinations of the head using the first clinically approved PCCT. The analysis was based on well-established objective image criteria, such as SNR of gray and white matter as well as CNR, in various regions of the brain. The findings are of clinical relevance since grey and white discrimination is one of the most overall challenging clinical tasks in CT since there are only about 10 HU attenuation differences between healthy grey and white matter, and various major health challenges, such as stroke, tumors, and inflammation, are to be depicted within cranial computed tomography.

The study shows, that the current PCCT PERs outperform MERs in terms of SNR of gray and white matter as well as CNR. However, the MERs performed better in terms of reducing beam-hardening artifacts below the cranial calvaria and in the posterior fossa between the petrous bones. In PERs, it is interesting that below the cranial calvaria there is a decrease of the signal due to the artifacts, although usually there is an increase of the signal. In this context, it must be emphasized that the currently available software version for the postprocessing of the data includes an algorithm for beam-hardening-artifact reduction for the MERs but not yet for the PERs. Thus, future software updates should improve the postprocessing algorithm for beam-hardening-artifact reduction in the PERs, especially since it is a novel ability of PCDs in comparison to conventional and dual-energy CT scanners that they allow for an accurate mapping of beam-hardening artifacts [20]. This mapping of beam-hardening artifacts could potentially be the basis for better artifact reduction in the future.

Several previous studies investigated the image quality of dual-energy CT systems (e.g., [14,18]). These studies usually focused on the MERs. Several groups demonstrated that the image quality of certain MERs in DECTs is superior to the quality of conventional polyenergetic images of a single-energy CT [16,18,21]. It has also been shown that artifacts can be reduced in DECTs using MERs [22,23]. Furthermore, Lennartz et al. showed that the use of MERs is associated with better detection of lesions in unenhanced CT [24]. In the future, it seems conceivable that the PCCT will be superior to the currently established DECT technology in terms of image quality.

In comparison to the results of the previous study by Neuhaus et al. that tested image quality of unenhanced examinations of the head performed with a dual-layer spectral CT (DECT) [14], it is relevant to point out that the PERs of the PCCTs seem to perform equally or better than the PERs of the referenced DECTs in regard to quantitative measures even though the radiation dose was lower (CTDI 44.07 ± 3.23 mGy compared to 55 mGy). For example, the SNR of gray matter in the present study (PCCT) was 20.1 ± 5.5, whereas it was 12.4 ± 1.7 in this DECT study. Likewise, the CNR in the present study (PCCT) is 2.90 ± 0.92, whereas it was reported to be 2.71 ± 0.39 in this DECT study. This difference may not represent the actual difference in image quality of PERs of the different detectors because various parameters for the image acquisition and calculation differ between both studies. However, this comparison may give a general indication of the PCCT potential, not only regarding high-contrast but also low-contrast soft tissue imaging. For example, the indices describing beam-hardening artifacts were stronger in this reported DECT study. Furthermore, in accordance with the clinical practice, axial reconstructions in the present study were made with a slice thickness of 3 mm, compared with 5 mm in the reference study (DECT) [14]. Noise, however, decreases with increasing slice thickness [25]. Despite the lower dose and slice thickness in the present study, the SNR and CNR of the images acquired with the PCCT are greater compared to the images acquired with the DECT, which may indicate the superiority of the PCD.

The quality of the MERs of the PCCT (Figure 5) is significantly different from those of the DECT and are inferior in every image-quality parameter. It has been described as early as 1987 that virtual monoenergetic images (MERs) can be created on the basis of the DECT [26]. Ultimately, MERs are the result of complex mathematical processes [27]. However, especially for MERs in the low keV range, increased noise is still a problem. The noise can be reduced by applying dedicated algorithms [28]. It is obvious that the experiences with these postprocessing algorithms are mainly based on data acquired with a DECT [28,29]. From the perspective of a clinical radiologist, it seems likely that there will be significant advances in the area of postprocessing of PCCT data in the near future. With improved postprocessing of the MER, it is then conceivable that the MER will be superior to the PER.

There are certain limitations of the present study. Firstly, the lack of a beam-hardening-artifact reduction algorithm for the PER limits the results of the present study. Secondly, the subjective image quality was not evaluated, which shall be subject to future studies as postprocessing algorithms improve. Thirdly, CT examinations with extensive pathologies were excluded in order to not influence the analysis of objective image criteria. Further studies investigating the performance of PER and MER for the diagnosis of dedicated pathologies, such as ischemic stroke, hemorrhages, tumors or edema, are warranted in this context.

For the clinical practice, the results of the present study suggest that the PER in combination with a MER for the assessment of the subcalvarial space and the posterior fossa are the best-performing reconstructions for reporting at the current state. When selecting the appropriate MER, a compromise between low SAI and PFAI and the highest possible CNR would have to be achieved, as is the case, for example, with the 75 keV MER.

The introduction of PCDs into clinical diagnostics is generally considered to be the next major breakthrough in CT technology [6]. The results of the present study indicate the superiority of PCDs in unenhanced examinations of the head compared to the most advanced CT systems (DECTs). At the same time, the results also reveal that there is still great potential for improvement, in particular regarding the postprocessing of the data provided by PCDs, to further advance image quality and diagnostic precision.

## Figures and Tables

**Table 1:** Comparison of image-quality parameters in polyenergetic and virtual monoenergetic reconstructions. Data are presented as mean ± standard deviation in Hounsfield units (HU); SNR and CNR are dimensionless. GM: gray matter; WM: white matter; SNR: signal-to-noise-ratio; CNR: contrast-to-noise-ratio; SAI: subcalvarial artifact index; PFAI: posterior fossa artifact index. The keV levels are chosen to represent the ends of the spectrum for signal and noise, the highest values for SNR and CNR for comparison of these in MERs and PERs, and both the spectrum and the lowest values for the SAI and PFAI because they are superior to the parameters of the PER.

Image-Quality Parameter	Polyenergetic Reconstruction	Monoenergetic Reconstruction	*p* Value
GM Signal	41.93 ± 1.78	40 keV: 42.41 ± 6.17 120 keV: 32.93 ± 2.07	*p* = 0.540 *p* < 0.001
WM Signal	33.37 ± 1.84	40 keV: 26.87 ± 3.99 120 keV: 30.92 ± 2.05	*p* < 0.001 *p* < 0.001
GM Noise	2.21 ± 0.53	40 keV: 5.09 ± 1.36 120 keV: 2.63 ± 0.69	*p* < 0.001 *p* < 0.001
WM Noise	2.07 ± 0.41	40 keV: 4.8 ± 1.07 120 keV: 2.53 ± 0.6	*p* < 0.001 *p* < 0.001
GM SNR	20.08 ± 5.5	70 keV: 15.59 ± 4.22	*p* < 0.001
WM SNR	16.77 ± 4.16	70 keV: 12.8 ± 2.47	*p* < 0.001
GM-WM CNR	2.90 ± 0.92	65 keV: 2.28 ± 0.77	*p* < 0.001
SAI	3.90 ± 1.48	40 keV: 9.82 ± 5.19 70 keV: 3.57 ± 1.22 120 keV: 2.49 ± 0.58	*p* < 0.001 *p* = 0.324 *p* < 0.001
PFAI	9.67 ± 3.61	40 keV: 13.84 ± 7.77 85 keV: 3.8 ± 0.82 120 keV: 4.31 ± 1.39	*p* < 0.001 *p* < 0.001 *p* < 0.001

## Data Availability

The data are available from the corresponding authors with reasonable requests.
